# New Method for Evaluating Surface Roughness Parameters Acquired by Laser Scanning

**DOI:** 10.1038/s41598-019-51545-7

**Published:** 2019-10-21

**Authors:** Leandro Tonietto, Luiz Gonzaga, Mauricio Roberto Veronez, Claudio de Souza Kazmierczak, Daiana Cristina Metz Arnold, Cristiano André da Costa

**Affiliations:** 1 Graduate Program in Applied Computing, Unisinos University, São Leopoldo - RS, Brazil; 2 VIZLab (Advanced Visualization & Geoinformatics Lab), São Leopoldo - RS, Brazil; 3 Graduate Program in Civil Engineering, Unisinos University, São Leopoldo - RS, Brazil

**Keywords:** Civil engineering, Computer science

## Abstract

Quality evaluation of a material’s surface is performed through roughness analysis of surface samples. Several techniques have been presented to achieve this goal, including geometrical analysis and surface roughness analysis. Geometric analysis allows a visual and subjective evaluation of roughness (a qualitative assessment), whereas computation of the roughness parameters is a quantitative assessment and allows a standardized analysis of the surfaces. In civil engineering, the process is performed with mechanical profilometer equipment (2D) without adequate accuracy and laser profilometer (3D) with no consensus on how to interpret the result quantitatively. This work proposes a new method to evaluate surface roughness, starting from the generation of a visual surface roughness signature, which is calculated through the roughness parameters computed in hierarchically organized regions. The evaluation tools presented in this new method provide a local and more accurate evaluation of the computed coefficients. In the tests performed it was possible to quantitatively analyze roughness differences between ceramic blocks and to find that a quantitative microscale analysis allows to identify the largest variation of roughness parameters *R*_*a*_*avg*, *R*_*a*_*sdv*, *R*_*a*_*min* and *R*_*a*_*max* between samples, which benefit the evaluation and comparison of the sampled surfaces.

## Introduction

The most traditional technique of vertical closure of buildings is masonry lined with coating mortar. However, coating mortars frequently present pathological problems such as adherence failures. Considering this situation, many studies have been carried out to understand the phenomena of adhesion between mortars and substrates. The adherence depends on the characteristics of the mortar, the substrate (such as its porosity and roughness, which are necessary for good interfacial interlocking), the application technique, and the climatic conditions during and after the application^1^.

Characteristics of brick and block layering surfaces such as pore size, pore size distribution and porosity play a crucial role in the adhesion between mortars and substrates. The surface texture influences the shear, and a smoother surface has lower adhesion strength^2^. Low adhesion and low adhesion strength are a major weakness of masonry. Adhesion strength depends on interrelated factors that may directly affect the bond development between substrate and coatings (e.g. surface water absorption, pore structure, mortar composition, mortar water retention and curing conditions) or indirectly (by surface texture and workmanship). While surface absorption characteristics define the rate and volume of water to provide mortar workability, the adhesion of the mortar is defined by the amount of water available at the interface and the strength of the hydration products deposited on the surface pores of the substrates. The required strength is not related to the mortar compressive strength, but by other criteria such as their workability, water retention and plasticity^3^.

It is agreed that the substrate roughness should be analyzed at two scales: the macroscale (on the order of millimeters), visualized in the form of protuberances, recesses, and superficial imperfections that allow mechanical anchorage on the surface, and the microscale (on the order of micrometers), where adherence occurs predominantly through chemical interactions between the atoms or molecules of the mortar and the substrate^4,5^. On this scale, there are few papers and several knowledge gaps. There is no known optimal parameter for each surface^6^. One of the greatest difficulties is the quantitative determination of the microroughness of substrates. Most of the studies make a two-dimensional determination of the roughness, thereby generating roughness coefficients that are not well correlated with the adherence.

Perez *et al*.^7^ analyzed the influence of surface treatments on the texture of concrete substrates. The authors used a 2D mechanical profilometer and found that the 2D method was not enough to characterize the surface, as already pointed out by Klingvall Ek *et al*.^8^, the same material may have different roughness depending on the measurement direction. Garbacz *et al*.^9^ says that laser profilometry generates a more detailed picture of the mechanical method that is used in 2D measurement and that laser profilometry parameters are 1 to 7 times larger than mechanical sampling. Sadowski *et al*.^10^ adopted the 3D profilometer (laser profilometer) to quantify the roughness of concrete surfaces, verifying that the method was satisfactory, since it presents a broadest surface reading when compared to the 2D method^11^. The shortage of literature in this area frustrate the comparation of the results found by the authors^12^. It has not yet been possible to estimate how much the texture vary along the surface, how this will influence the adhesion of the coating to the substrate and which is the best method for characterizing each substrate in construction. Grigoriadis^13^ for computing parameters of 3D data, calculate the roughness, generating the average value between the roughness values of the lines that make up the surface, such as a 2D analysis, without an analysis of the surface as a whole.

Three-dimensional (3D) evaluations of roughness are unusual and are typically presented as figures representing the surface topography^14^, but these are not quantitative, which results in an empirical specification of a parameter representing the roughness of the substrate and generates a roughness coefficient that cannot be extrapolated to other surfaces.

The most common form of analysis of these materials is manual and subjective^6,11^, whereby the surface is analyzed and evaluated based on the knowledge and skill of a professional. However, there is equipment and software that help to inspect the surfaces of materials. From a light detection and ranging (LiDAR) technique, for example, with the use of a 3D laser scanner, it is possible to acquire a number of points with sufficient resolution, called a point cloud, and their corresponding geometric surface, to evaluate the irregularities on material surfaces. With the irregularities generated from points with 3D coordinates, it is possible to determine the salient features of surfaces, which allows civil engineers to determine the quality of adherence to other materials. These artifacts of valleys and peaks (relief features) on a surface are called roughness. Therefore, the roughness of a given surface is evaluated to determine the interface quality thereof with other materials, and especially, as already mentioned, with concrete.

To perform this subjective evaluation assisted by software, the surface reconstruction is performed from the point cloud. Several works^15–18^ in the area of reconstruction of surfaces have been proposed, each with its purpose or application context. Surface reconstruction aims to analyze the individual information of the points and reconstruct as much information as possible about the scanned objects, thus allowing the visual analysis of the surface protrusions.

However, visualization-based measurements use purely geometric techniques for data manipulation and are entirely subjective and, therefore, subject to the skill and experience of the professional involved. Another important issue is the lack of a standardization of evaluation. Precisely because of its subjective nature, a professional could determine one evaluation and another, although with similar results, could determine a different result for the same evaluated area.

Toward solving this evaluation problem, techniques for evaluating coefficients or roughness parameters are used to quantitatively measure the interface quality of a surface. From these measurements, it is possible to define the overall roughness of the surface in a standardized way, and thus allow a more accurate evaluation of the surface interface of the material.

Although the measures suggested in the literature are adequate to measure material roughness, they are performed over a given area, as a whole. In this way, an average or smoothed value is obtained for the surface, and no local evaluation is considered. In this sense, an analysis with a spatial subdivision of the surface could provide a more precise and detailed analysis of the surface adhesion in certain places. In addition, it would be possible to evaluate the standardization of similar surfaces, considering that the sampled surfaces of the same block should be similar, owing to the material properties and the physical conditions to which the block was subjected (temperature, pressure, etc.).

In this work, is proposed a technique that performs an analysis of a point cloud acquired by laser scanning and computation the roughness parameters at different scales on a hierarchical structure of the spatial division of data. From the fitting plane representing the point cloud and keeping the data in a hierarchical data structure, more specifically a spatial division tree (a quadtree), the parameters or roughness coefficients are computed for all locations, at all levels of spatial division. In this way, the surface roughness is measured both at the global level (higher level of the tree) and local level (lower level of the tree).

Our hypothesis is that from the local evaluation, it is possible to better evaluate the parameters of roughness by location and in this way to better estimate the level of adhesion of the materials, in a localized way. In addition, it is estimated to identify in a standardized and quantitative way the similarity between surfaces of sampled materials. Surfaces that have similar roughness coefficients in different subdivided regions, in equivalent positions, indicate the same manufacturing context (material, temperature, pressure, etc.) and have a better evaluation of the standardization of blocks or parts of the same material.

## Related Works

To compute roughness coefficients or parameters to evaluate a surface, it is necessary to obtain the data that form the sampled surface. Computer hardware and systems are used for the computation of surface roughness. An efficient way of obtaining surface information is through laser scanning equipment^15,19^. In this technique, a ray emitted by the equipment hits the target, and its reflection is read by the equipment to measure the position and depth of the point where the ray collided with the target. In some equipment, the color associated with the hit point is returned. The result of this sampling is a point cloud. From the point cloud, the geometry and measurements are computed that relate the points with the fitting plane of the surface by a mean least squares method.

The analysis of the roughness and salience of the sampled surfaces are used to evaluate their quality. Some works^16–18,20–23^ have performed the visual evaluation of surfaces based on geometric analysis to determine the roughness and salience measurements. In this type of evaluation, in general, the surface is reconstructed from a point cloud, generating a polygonal mesh. The surface reconstruction from sampled points is a well-studied problem in computer graphics^15^. These approaches^16–18,22,23^ can obtain good results for geometric surface reconstruction and for the qualitative evaluation of surfaces.

The approaches used are triangularization and volumetric methods. In triangularization as presented in^24–26^, the algorithms search for neighboring points in a certain direction to form triangles and, from the set of triangles, obtain a polygonal mesh. In^24^ authors define Delaunay based mesh triangulation as the geometric dual of the Voronoi diagram, so from the Voronoi diagram, sites are defined as vertices of triangles and neighboring cells are connected to form triangles. In^25^ the authors use triangulation approach by inserting an energy term for Delaunay tetrahedra problem, ensuring greater robustness of the method over mesh noise. Wang *et al*.^26^ work over an unoriented point cloud using Delaunay tetrahedron and get better results in smooth surface reconstruction. After obtaining triangles via 3D Delaunay triangulation, a good initial triangle is considered to be the seed of the mesh and from it other appropriate triangles are connected to their front edges, those that are not connected to any other triangle. The initial triangle is the one that forms as flattest surface as possible with its adjacent ones. And so the mesh grows iteratively for all front edges until there are no more suitable candidate triangles. Suitable triangles are those that have edges that close with the current triangle and its neighbors on front edges and with an angle smaller than a threshold parameter. These methods generally reconstruct smooth surface and either incorporate roughness as a mesh relief (not treating it as non-mesh points) or remove it as a noise from the points.

The most popular volumetric methods^16–18,27,28^ are available for authors and are used in commercial software. These methods aim to obtain a surface *S* that is formed by *N* ordered points of a point cloud, where the set of points *D* is *D* = (*p*_1_, *n*_1_), …, (*p*_*N*_, *n*_*N*_), each *p*_*i*_ is a specific sampled point, and each *n*_*i*_ its respective normal. The formal surface definition is *S* = *x* : *f*(*x*) = 0. The main algorithms of surface generation through volumetric methods are “smooth signed distance” (SSD)^18^, “Poisson surface reconstruction”^16^, and^17^.

The “Poisson surface reconstruction” algorithm^16^ obtains a model indicator function (an implicit function), where the gradient of this function is a vector field that is zero at almost all points, except for points near the surface, where the value is equal to the normal of the points sampled. Thus, the algorithm looks for the gradient function that best approximates the local vector field (direction), associated with each point. This algorithm is ideal for use in the context of simplified point clouds for surface visualization, because it is a global solution that involves all the data, generates smoothed surfaces, and is consistent (robust) to work with discontinuities or noise in the data, which happens in the point cloud after a simplification process^16^. It is one of the most popular methods for surface reconstruction owing to its scalability and efficiency^29^. Therefore, it is suitable for surface reconstruction with a focus on visualization and does not favor the analysis of surface roughness.

A known problem of the method^16^ is the excessive smoothing of the surface^17^. In this sense, the SSD^18^ and screened^17^ algorithm deal with this problem using positional constraints on^17^ the optimization and the gradient function^18^.

From this geometric data, the roughness and salience are computed. In^20^ the computation of the salience coefficients is performed by comparing the height of the vertices in a given region around a vertex (neighboring vertices). Defining the quality of the model by the shape of the rough area is a task of subjective and relative perception; for example, the size of the rough area also depends on the measurement of the size of the model. Natasha *et al*.^11^ also describe how difficult it is to distinguish roughness from salience when evaluating a geometric model. In addition, other problems are related to the evaluation of geometric surfaces, mainly because they are polygonal approximations. These methods are suitable for viewing and not for a proper roughness measurement.

Recent works^6,11,30^ focus mainly on the quantitative assessment of surfaces roughness (called roughness parameters). From the computation of these parameters, it is possible to standardize the evaluation of the sampled material surfaces. These measurements are described in the literature^6,30–33^ and are used to measure the level of adherence and quality of the material surfaces according to their roughness.

The main roughness parameters reported in^6,11,30^ are *average roughness* (*R*_*a*_) and *root-mean-square roughness* (*R*_*q*_). These measures evaluate the average standard deviation of the heights (valleys and peaks) in a surface profile to compute the degree of roughness. However, for the computation of these parameters, it is first necessary to compute the fitting plane for the points acquired from the surface. From the plane coefficients, it is possible to determine the height of a peak or valley by evaluating the height coordinate of each point of the cloud. The calculation of the plane is described in more detail in Section 4.1.

The *average roughness R*_*a*_, described in^6,30^, is given by:

$${R}_{a}\approx \frac{1}{n}\mathop{\sum }\limits_{i=1}^{n}|{z}_{i}|$$

where *z*_*i*_ is the height coordinate of the current point. The *root-mean-square roughness* (*R*_*q*_), also described in^6,30^, is defined as:

$${R}_{q}\approx \sqrt{\frac{1}{n}\mathop{\sum }\limits_{i=1}^{n}{z}_{i}^{2}}$$

**Figure 1:**
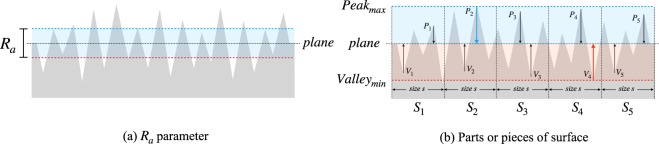
Surface profile described in^6,30^, with peaks and valleys. (**a**) The value of the parameter *R*_*a*_, and (**b**) dividing the surface into parts to compute *R*_*a*_. Based on the images presented in^6^.

Figure 1, presented in^6^, illustrates the behavior of the parameter in relation to a profile of a sampled surface.

However, Santos *et al*.^6^ also point out that the *R*_*a*_ and *R*_*q*_ parameters do not provide any type of local surface evaluation. For a local measurement, other roughness parameters are used, based on the division of the surface profile into smaller parts and considering information on peaks and valleys separately. In this way, it is possible to analyze a greater level of detail about the roughness evaluation. These parameters are *mean peak height* (*R*_*pm*_), *mean valley depth* (*R*_*vm*_), *mean peak-to-valley height* (*R*_*z*(*DIN*)_), *ten points height* or average of five peaks (*R*_*z*(*ISO*)_), *maximum peak height* (*R*_*p*_), *maximum valley depth* (*R*_*v*_), *maximum peak-to-valley height* (*R*_*max*_), and *total roughness height* (*R*_*y*_), which is the sum of the heights of the highest peak and the deepest valley.

The main feature involved in the computation of these parameters is that they are obtained from samples/patches of a surface, providing a level of local control, because the maxima and minima of each part are considered. Figure 1(b), presented in^6^, shows the relation of the calculated parameters on peaks and valleys with samples (or patches) of the surface profile.

Finally, despite the local control provided in the calculation of the parameters based on the division of the profile into patches/samples, in^11^, the authors also indicate that multiresolution surface analysis yields the best results for roughness computation from geometry. Other works such as^23,34,35^ do not aim at quantitative roughness measurement, but use a roughness measurement as a subjective evaluation criterion of mesh reconstruction quality. In this work, is proposed a spatial division control that allows analyzing the sampled surface at hierarchical levels (described section 4.1.2).

## Method for Point Cloud Acquisition

**Figure 2:**
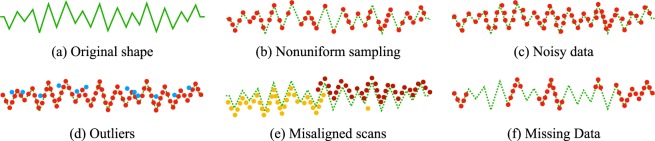
Types of failures found in point cloud, based on the images presented in^15^. In the tests performed, more frequent problems were found with (**b**) nonuniform sampling, (**d**) outliers, (**e**) misaligned scans, and (**f**) missing data. These problems are covered in more detail in^15^.

The success of cloud computing is directly related to the quality of the input data. It is common for reading and, in this case, laser-scan (LiDAR) equipment to produce a cloud of erroneous points^15^ (see Fig. 2), which can lead to computation errors, either in the roughness computation or in the reconstruction of 3D surfaces. In the case of surface reconstruction, the main works use several techniques to treat the cloud point imperfections to solve or reduce the problem, either with input data constraints, the type of geometric form treated, or related to the type and shape of the computed output data^15–18,22^. However, for the computation of roughness coefficients, this is not possible. Only valid information is handled, and thus the more data is acquired, the better is the achieved accuracy.

Several factors can impact the final result. Throughout the process, several faults can occur, from the preparation of the material to the reading of the data by LiDAR. Imperfections in the preparation of surfaces, errors in the preparation and firing of the material, errors in the surface preparation for reading, surface cleaning, artifacts produced by markings on blocks, the orientation of blocks, surface unevenness, and regulation (height x accuracy) on reading. These error factors can be controlled or avoided throughout the process.

### Blocks production process

**Figure 3:**
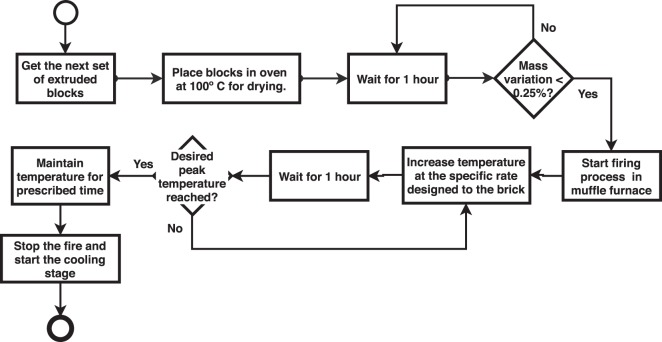
Blocks production process. Each block must be carefully prepared for the reading process.

The blocks production process is carried out in two steps, the block conformation in the production line in brickyard and the ceramic firing cycle in different temperatures. The data acquisition of block should be done on samples that do not contain imperfections, such as: grooves, broken pieces or others caused by packing or transportation. If it is not possible to satisfy this premise, it is recommended to follow the burning process as shown Fig. 3, aiming to ensure a few imperfections and homogeneity between blocks, favoring the process of reading data.

The firing process of blocks is defined as:

1. Separate all extruded blocks that will be used for the same firing temperature.
2. The blocks should be placed in an oven at 100 °C for drying, which is achieved when the mass change within one hour was less than 0.25%.
3. When the stabilization is achieved, the firing process starts. The muffle furnace is heated to rate designed to the brick.
4. Every hour, the temperature should be increased at the rate designed to the brick until it reaches the desired peak temperature.
5. The blocks must be maintained at peak temperature for a prescribed time.
6. The muffle furnace is turned off and the block is allowed to cool.
7. Cut a face for use in the reading process.

In this process, it is important to pay attention to the handling of the blocks to prevent artifacts from being produced and causing failure as illustrated in Fig. 2, mainly because outliers can be produced throughout the process.

### Surface reading process

The purpose of the reading process is obviously to obtain as many points as possible from a^15^ surface. It is a relatively simple process, but requires some basic procedures to ensure an effective reading method. It is necessary to essentially set the position and size of the region of interest and the number of points to be read, adjusted with respect to the reading accuracy of the equipment. The higher the accuracy, the better the quality of the information, and thus it is important to adjust the equipment for maximum reading accuracy.

**Figure 4:**
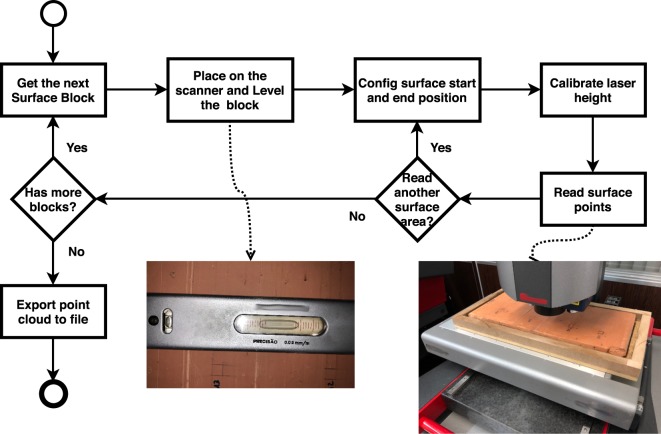
Process for reading the surfaces.

Figure 4 illustrates a process to successfully read data, avoiding problems due to several factors associated with the equipment and, especially, the sampled objects.

The focus of the reading process the proposed method, however, is the description of the activities necessary for the acquisition the point cloud for surface roughness analysis, and it does not contain steps or details of activities for processes that acquire point clouds with other objectives. For example, was considered in this process a tabletop scanner where the part to be scanned rests on a table and the equipment emits a laser beam from top to bottom. Additionally, was not considered the acquisition of other information that may be relevant to the reconstruction of surfaces, such as normals or colors, because the process focuses precisely on the information of the points and especially the height (*Z*-axis) to calculate the roughness.

The process that is performed after the parts acquisition is illustrated in (Fig. 4):

1. For each acquired surface block, the level of the block in the equipment must be adjusted to avoid problems caused by the orientation of the part (in Section 3.2.1, the problems related to the part rotation are described).
2. Set the surface scanning location. At this point, must choose places that do not have artifacts or problems in the material surface. One should avoid scratches, reliefs produced by the materials that make up the surface, small holes, great depressions, and imperfections in general. A good approach is using a template of the desired surface size to mark the starting position of a region of interest and then manually set the scanning end position in the machine software. In this way, the equipment will read a rectangular region aligned with the axes of the plane of the surface base (*XY* plane).
3. Adjust the equipment for better reading accuracy depending on the height of the block in relation to the equipment. LiDAR equipment generally allows adjustment of the height of the laser via software and hardware, and one can manually set the reading accuracy with respect to the height.
4. Set the number of points to be read (number of rows and columns), check the final number of points that can be read and whether the reading accuracy (minimum distance between points in the *X* and *Y* directions) is in agreement with the accuracy of the equipment. Start the reading process of the LiDAR equipment.
5. For each surface sampled, repeat the steps from item 2.
6. At the end of the process, export from the equipment software the scanned point clouds for processing in the roughness calculation software.

#### Settings and problems related to reading data

The most important configuration in this process is the reading accuracy in the height direction (treated as the *Z*-axis, by default). This adjustment is defined by the equipment software, which must have a means of calibrating or adjusting the height for reading the data to achieve greater accuracy of points. If the laser height becomes inadequate, the machine may not read as many points as are configured for the defined surface or have a read error (such as an example in Fig. 2). In addition to the problem of reading sensor height and reach, high light absorption and occlusions in the scanning process may result in a great loss of reading information^15^.

It is important to emphasize that because the surface blocks have height variations, owing to the manufacturing process of the pieces and possibly exacerbated in the firing process, the height adjustment must be performed for the block and also for each surface to be digitized.

In the reading process of the LiDAR equipment, failures occur mainly because of artifacts or imperfections of the blocks. However, reading problems may also occur if the surface is not well positioned and oriented. The positioning and rotation of the block relative to the base does not significantly interfere with the process or cause read failures. However, the alignment of the surface with respect to the axes of the base plane (*XY* plane) favors the reading, standardization, comprehension, and treatment of the information. The relevant problem during scanning is the rotation of the block in height relative to the plane of the scanner base. In this sense, if the block has a “vertical” orientation, the laser can fail in two situations: height calibration (*Z*-axis of the scanner) that is not adaptive to any-place read, because the laser is calibrated to a global height during reading and considers only slight variations in height along the surface. This sharp local height difference can lead to equipment readability failure, and the other point of failure is a “shadow” effect. Although unusual, this occurs when one point prevents the reading of another point because it exerts an occlusion on the view of this other point by the laser beam.

The vertical rotation problem is caused by artifacts and a failure to cut the blocks during the acquisition process. To avoid the rotation problem and benefit the acquisition of as many points as possible on the surface, the block must be aligned to the axes of the scanner base and arranged in support that allows leveling. This leveling removes any vertical surface orientation problems. To ensure this, the block must be placed under a material that allows molding or leveling at reading time. In Section 3.3, is described how to solve the problem of leveling and rotation of blocks.

Finally, it is important to note that many problems that cause misalignment, lack of data, or noise are caused by the inherent characteristics of the equipment used. Therefore, because the results vary according to the equipment used, it is important to choose equipment that has the highest reading accuracy possible. However, it issues such as the noise level and the ability to configure and adjust the equipment should be considered.

### Adjustment of block leveling

During the tests, certain challenges were encountered (as illustrated in 2) in ensuring the effectiveness of the process of reading the point clouds of the surfaces.

When scanning a sample, readability problems can occur owing to improper leveling (see Section 3.2.1). To solve the problems of rotation and leveling, an apparatus was developed to facilitate the handling and leveling of the blocks. The system must support the block and allow the position and level adjustments. The dimensions should be adequate to avoid overweight and facilitate the handling of the block. It should be ensured that the mass of the assembly does not exceed the limit specified for the LiDAR equipment used.

**Figure 5:**
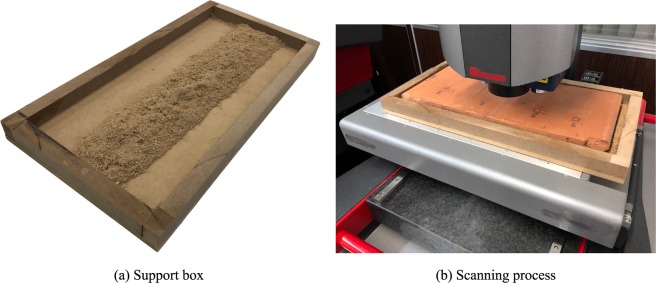
Support box (**a**) for position adjustment and part rotation. Scanning process (**b**) with the support box.

Figure 5 shows the support box used in the tests, fabricated with wood, with 25 mm height and horizontal dimensions suitable for the sample, plus a clearance of 10 mm. It is important to note that the read settings of the equipment must be configured according to the specifications of the equipment used. The process steps must be set so as not to impair the operation of the equipment or the reading of data.

## Surface Roughness Computition

**Figure 6:**
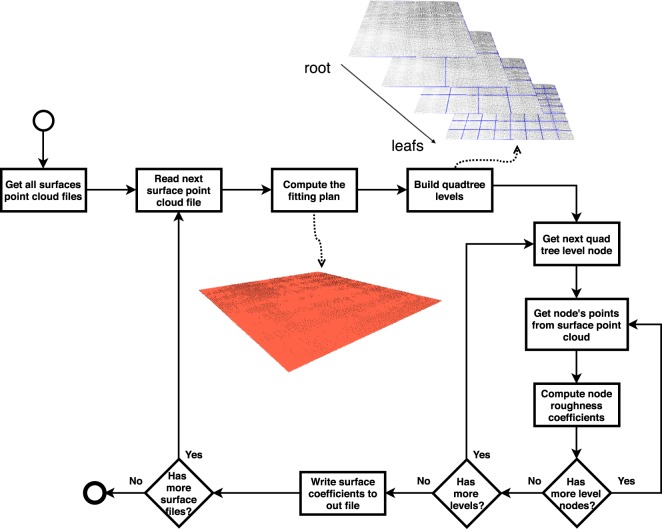
Process for computing surface roughness coefficients.

After the point cloud acquisition process, the process of computing and representing of the roughness coefficients of the surface as a quadtree is executed. The computational process of the roughness coefficients is shown in Fig. 6.

The computation process of the roughness coefficients must be performed for each point cloud of the surfaces read in the reading process:

1. Read the next cloud point file (one per scanned surface). The data are exported and must be translated into a 3D vertex structure.
2. The fitting plane is computed, as described in section 4.1.
3. The quadtree must be set up according to the number of levels defined for the program. See Section 4.1.2 for details.
4. The coefficients are computed by level and by node. Therefore, for each node and each level of the quadtree:
  - Get the points belonging to the current node’s region.
  - Calculate the roughness coefficients as described in Section 4.1.1.
5. The roughness coefficients computed for the surface are written in the output file for further visualization and processing.

### Fitting plan computation

Roughness measurements are essentially height differences relative to the average height of a cloud of points at a given location or fitting plane. To perform this computation, is defined the plane that best fits (represents) the points cloud. This plane should consider the orientation and slope of the surface as sampled. Through the calculated plane, it is possible to evaluate the height difference of any point of the cloud in relation to the height of the estimated surface (i.e., in relation to the height of the plane). The equation that defines the plane (considering the Z-axis as height) is:

$$Z={b}_{0}+{b}_{1}x+{b}_{2}y$$

To calculate the fitting plane, the *least squares product* technique is used, whose objective is to find the coefficients of this support plane from the points of the surface. From the coefficients, it is possible to determine the height of the plane in relation to any point on the surface. The matrix *B* that defines the coefficients of the plane (*b*_0_, *b*_1_, and *b*_2_) is given by:

$$B={({A}^{T}\times A)}^{-1}\times ({A}^{T}\times L)$$

where *A* is the matrix formed by the points of the surface without the height coordinate (in this case *z*), *A*^*T*^ is the transposed matrix of *A*, and *A*^−1^ is the inverse matrix of *A*. Finally, *L* is the matrix composed by the coordinate *z* of all the points in the cloud. The matrices *A* and *L* are defined as:

$$\begin{array}{cc}A=[\begin{array}{lll}1 & {x}_{0} & {y}_{0}\\ 1 & {x}_{1} & {y}_{1}\\ \vdots  & \vdots  & \vdots \\ 1 & {x}_{n} & {y}_{n}\end{array}] & L=[\begin{array}{l}{z}_{0}\\ {z}_{1}\\ \vdots \\ {z}_{n}\end{array}]\end{array}$$

**Figure 7:**
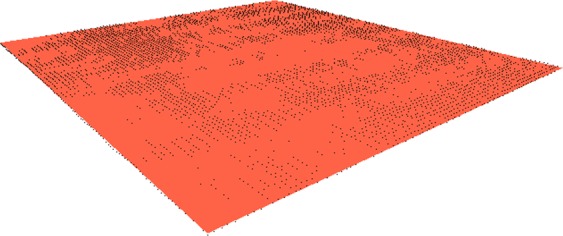
Example of a fitting plane (in orange) computed on a cloud of points (in black).

Figure 7 shows an example of a fitting plane that has been computed through its coefficients (matrix *B*) and the points at the corners of the surface.

#### Computing the roughness parameters

The roughness parameters are computed from the coefficients calculated for the plane, by calculating the average roughness (*R*_*a*_), considering that *z*_*i*_ is the height and it is calculated as *z* = *b*_0_ + *b*_1_ × *p*_*i*_*x* + *b*_2_ × *p*_*i*_*y*. The *z* calculated for *R*_*a*_ is absolute; however, to calculate peaks and valleys, positive and negative values are considered in relation to the *z* of the plane. This non-absolute value is the basis for the computation of the other roughness parameters, whose formulations are presented in^6^ and mentioned in Section 2.

Another important question of implementation is the division of the surface area or part of it into samples or patches. Because a 2D profile is considered for the calculation, an area division parameter is defined to consider a configurable number of patches or sub-areas. In the performed tests, this parameter is defined as 5, as described in^6^. Although it is possible to explore other values, in this work it was not considered relevant to perform experiments with other values.

#### Hierarchical structure for roughness parameters representation

Coefficients or roughness parameters, calculated over the whole surface, indicate the average or overall values for the entire sample region. However, the local distribution of these values is not considered. It is common for a surface to introduce a coefficient variation according to the local sampling of roughness.

The evaluation of the parameters in a specific location, respecting a location criterion, benefits both the comparisons among the different regions of the surface and among the several samples of a larger material surface or of several pieces produced with the same material. It is expected, for example, that significant differences occur on the surface of a sample. However, among different samples of the same fabrication, similar behavior is expected for the coefficients of the same locale or within an expected variation due to possible changes in the surface confection process.

To evaluate the roughness parameters at several locales of a material sample, a hierarchical spatial division of the sampled area is proposed. Although this spatial division can be implemented in several ways, it is relevant to consider a pattern of area and location for each region and the size of the region itself. A pattern and location make it easy to compare different samples and the size of the region is important to determine the significance of the roughness measurement.

The size of each region can vary significantly among different material types. Therefore, this is a parameter of the size that must be defined by the user, who evaluates the computed roughness values. To benefit the spatial division (location and size) and its manipulation, a quadtree data structure is adopted.

**Figure 8:**
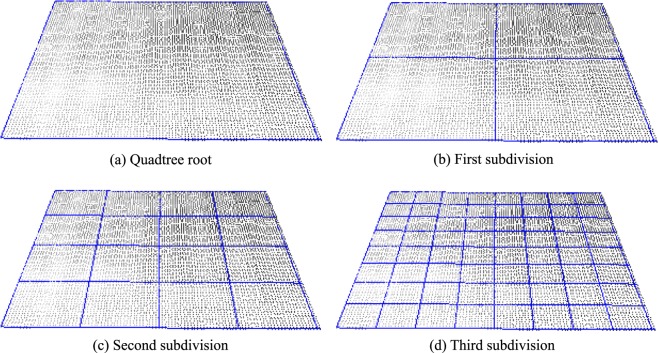
Hierarchical spatial subdivision with a quadtree. (**a**) Root element; (**b**–**d**) successive subdivisions.

A quadtree is a widely used structure for a spatial division to represent scenarios in computer graphics, whether for collision testing, representation of structures for a level of detail, or for maintaining hierarchical information about a particular location. As a characteristic, a node of this type of tree can be a leaf (without children, that is, the end of a branch) or a branch (having exactly four children). A tree branch node has average values that represent the calculated values of its four children. If the children are also branches, then they also have average or representative values for their four children and so on. Thus, at the highest level of the tree (called the root) are the have the roughness coefficients computed for the entire surface. At each level of the tree, the roughness parameters of each region are calculated with respect to the spatial division. The last level is the parameter defined by the user for the minimum area of evaluation of the roughness parameters. Figure 8 shows an example of different levels of division of the surface using a quadtree.

#### Computing roughness parameters for quadtree levels

After the spatial division of the surface, according to the coordinates that form the surface polygon, the classification of points by region/division is executed. This process is recursive; that is, the points are also classified within the region of the successive subdivisions.

It is relevant to note that the roughness parameters of the different branches of the tree are not obtained by average; they are recalculated, which determines the better accuracy of the parameters. For each spatial subdivision, the points of each region are considered for calculating the roughness parameters (as described in Section 4.1.1) and the values are saved in the tree structure. In this way, the user can evaluate the roughness coefficients at the various subdivisions.

## New Method for Surface Roughness Evaluation

**Figure 9:**
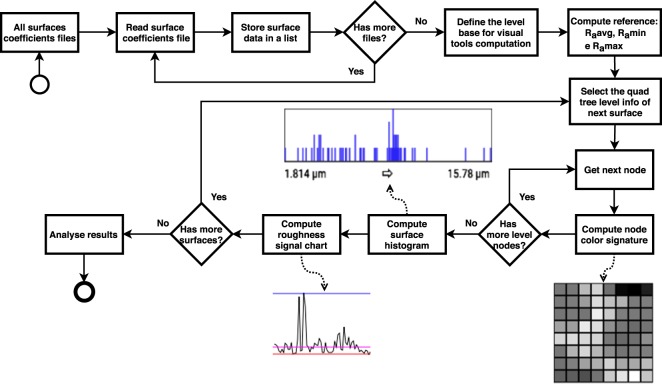
Process for computation of tools for evaluation and analysis of roughness coefficients.

After the computation of the roughness parameters at different levels of the tree, the information for representation and analysis of the results are processed in other software. Figure 9 presents the computation of the information generated by this new software for the analysis and evaluation of the roughness of the surfaces sampled.

The process of generating graphical information for analysis and roughness evaluation is defined as:

1. All files of the roughness coefficients (one per surface) are read by the software for analysis.
2. The surfaces are kept in a list for processing together.
3. According to the level of the quadtree defined for analysis, all nodes of all surfaces are traversed to determine the values of the *R*_*a*_ reference: medium (*R*_*a*_*avg*), minimum (*R*_*a*_*min*), and maximum (*R*_*a*_*max*). More details are described in Section 5.1.
4. For each node of each surface and according to the level of the selected quadtree:
  - Roughness codes or signatures are computed as defined in Section 5.1.
  - The node histogram is computed (Section 5.1.1).
  - A signal is assembled from the *R*_*a*_ data of the node and plotted on a chart comparing is with the reference values (Section 5.1.1).
5. A page with all the generated graphical objects is displayed to the user (civil engineer), who can evaluate and analyze measured roughness on each surface and infer their behaviors and the possibilities of adhesion in each situation or configuration of the block acquisition process.

### Surface roughness signature

**Figure 10:**
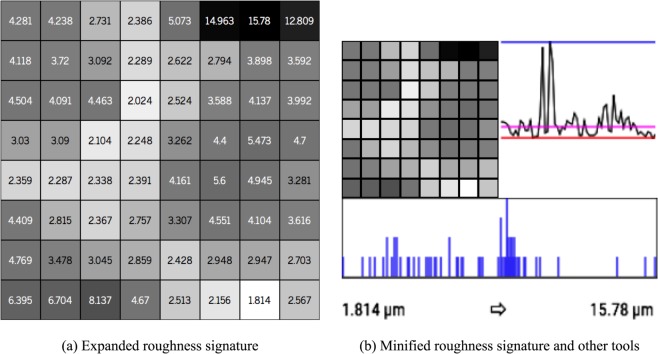
(**a**) Example of surface roughness code (signature). Computed from 4th level (3rd division) of quadtree roughness parameters (*R*_*a*_). (**b**) Example of surface roughness evaluation metrics. Center: visual minified code of the surface. Below: histogram computed from the visual code. Right: chart comparing the surface *R*_*a*_ coefficients with the *R*_*a*_*avg*, *R*_*a*_*min*, and *R*_*a*_*max* reference values.

A code or signature is created for surface, composed by the roughness values of each region defined by the subdivision of the quadtree. In this way, it is possible to visually identify the pattern of the sampled surface and compare it with another surface by its roughness signature. Figure 10 shows an example of the visual code generated from the reference coefficients (named the average roughness of the surfaces sampled *R*_*a*_*avg*) and the level of greater detail used for example, in the case the fourth level of the quadtree (3rd subdivision).

To generate the colors of the code (the visual signature), a gray scale is used, with the value of *R*_*a*_ local being compared with the mean (*R*_*a*_*avg*), minimum (*R*_*a*_*min*), and maximum (*R*_*a*_*max*) values of the reference. For comparison purposes and in the absence of a standard reference value in the literature, the mean of *R*_*a*_ (*R*_*a*_*avg*), as well as the minimum value (*R*_*a*_*min*) and maximum (*R*_*a*_*max*) of *R*_*a*_ computed on all the surfaces sampled are defined as reference values.

From these reference values, the closer the *R*_*a*_ location is to *R*_*a*_*min*, the closer to white the color will be. The closer the *R*_*a*_ location is to the *R*_*a*_*max*, the closer to black the color will be. Consequently, the closer to *R*_*a*_*avg*, the closer to gray-medium the be the color.

The maximum defined depth level (the least evaluated detail of the quadtree) is a user parameter. For the tests executed, the fourth subdivision level (1.5625 mm^2^) is used to exemplify the signature generation of the block, because it is not that far from the reading accuracy of the LiDAR equipment (0.1 mm) and with a reasonable number of points (≈156 points) for a range of *R*_*a*_ values.

#### Other tools for roughness evaluation

In addition to the visual signature of roughness, are also proposed the use of other metrics to evaluate and compare roughness (*R*_*a*_) among surfaces. Figure 10 gives an example of the metric set used for roughness evaluation and comparison.

First, from the color composition of the visual code, a histogram is built to check the frequency of the colors and compare the dispersion or concentration within the range of values *R*_*a*_*min* (white) and *R*_*a*_*max* (black). The *x*-axis of the histogram represents the 256 possible values of gray tones, white (*R*_*a*_*min*) to black (*R*_*a*_*max*). The *y*-axis represents the number of times (frequency) that a certain shade of gray appears in the code. The histogram can be used to verify the dispersion of the coefficients *R*_*a*_ with respect to the mean (*R*_*a*_*avg*) for each surface. The surface signature (grays tiles) allows a visual evaluation of the dispersion of the coefficients, and the histogram allows a quantitative evaluation of this dispersion.

Another form of evaluation for comparison of the surface coefficients with the reference parameters through a line chart, with which it is possible to verify the behavior and distribution of the coefficients of the surfaces in relation to the reference parameters.

To construct the chart, the array of the surface coefficients is transformed from two-dimensional signal into a one-dimensional signal. In addition to the coefficients, the reference values (*R*_*a*_*avg*, *R*_*a*_*min*, and *R*_*a*_*max*) are also plotted on the chart and, in a general way, the variations of the coefficients of the surfaces are evaluated. In this way, is possible to compare one surface with the others, verifying those that have greater or lesser variation of the coefficients in relation to *R*_*a*_*avg*.

In Section 6, are described the tests performed, the results obtained, and how to use these tools to evaluate surface roughness.

## Testing and Analysis of Results

In order to validate the proposed method for analysis and evaluation of surface roughness, roughness coefficients were computed and analysis information was generated for samples of red ceramic blocks. The objective of these tests is to verify whether the proposed techniques are satisfactory for the surface roughness evaluation of the blocks, allowing the association between this property and the adhesion resistance of coating mortars with the blocks. To this end, ceramic blocks were produced from the same clay subjected to firing cycles of 800 °C and 1000 °C, resulting in blocks with significant differences between their physical and mechanical properties.

The blocks were prepared, read, and analyzed according to the processes defined in sections 3 and 5. Finally, the visual information was obtained for analysis and evaluation of the results.

**Figure 11:**
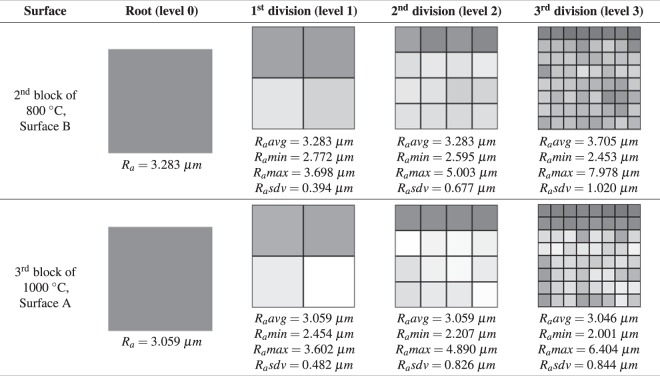
Surfaces roughness signatures in four levels of detail. In first column, the general *R*_*a*_ is showed bellow each figure. In other columns are showed mean (*R*_*a*_*avg*), minimum (*R*_*a*_*min*), maximum (*R*_*a*_*max*) and standard deviation (*R*_*a*_*sdv*) for each set of *R*_*a*_.

For the computation of roughness signatures, the 3rd level of subdivision of the quadtree hierarchy is used, because it is a good resolution for calculating roughness, as it approaches the reading accuracy level of the laser and it was the first division level that allowed us to verify a significant difference between the surfaces (see Fig. 11). To view the signature, a minified version of the figure (without the values of the coefficients *R*_*a*_) is defined, because it facilitates the visual interpretation and comparison of the results.

### Evaluation of the results obtained

To evaluate the results, the visual signatures of all the surfaces are compared. The other information (histogram and roughness graph) are also used to aid in the analysis of the coefficient behavior. Thus, in addition to the values *R*_*a*_ calculated for each location of each quadtree precision level (representing the data for quantitative assessment), as well as computed in the reference works^6,11^, this work presents new tools that allow a more accurate evaluation of the behavior of the coefficients over a surface and also comparisons of coefficients (signatures) between different surfaces.

In relation to the local evaluation, i.e., the visualization of the roughness information on the surface, with spatial separation of regions (quadtree), the roughness signature of the method allows a better interpretation of the locally calculated coefficients than the reference methods, described in^6,11^. One can better infer what happens to the internal coefficients and their distribution along the surface.

From the analysis of the signature, it is possible to visualize parts of the surface that have a greater variety of coefficients and to identify which parts have a greater (or lesser) level of roughness. Compared to the methods presented in^6,11^ there is a considerable gain in this respect, because the methods present only global roughness coefficients of the surfaces, without a local or detailed analysis.

Compared to the methods of surface reconstruction^15–18,21,22^, the proposed method has an advantage both because the information in these reference works is smoothed in relation to the original surface, and in the fact that it does not have the focus on the analysis of roughness coefficients, precisely because it aims to reproduce surfaces and not analyze roughness. These methods allow only a qualitative assessment of the roughness coefficients.

The roughness signature also allows an overall surface evaluation, comparing one surface with the other surfaces of the same block and with all surfaces sampled at the same firing temperature. This allows evaluation of the behavior of parts (surfaces) within the block itself, to find behavioral patterns that indicate a higher interfacial adhesion factor in certain locations and the global behavior of the coefficients in relation to blocks of the same temperature. For example, there are regions of a block that have greater roughness and others with lower roughness; it is possible to identify regions where the blocks could favor adherence.

In order to evaluate surface roughness coefficients, are proposed the analysis of both the detail levels of the quadtree divisions and the comparison between surfaces by analyzing the developed evaluation metrics (signature, histogram, and roughness graph).

#### Evaluation by level of detail

The first form of evaluation proposed is the hierarchical and local analysis of the roughness coefficients. In this evaluation method, it is possible to analyze and compare surface signatures at different levels of detail. The higher the level of division of the quadtree evaluated, the greater the level of precision of this evaluation, precisely because the previous levels are of global or average values in relation to a region. It should also be considered that in the initial level of the quadtree (root level) the calculated coefficient is the global value of the surface, that is, the same type of result as the works presented in^6,11^. Figure 11 presents a comparison at the initial levels of the quadtrees of two surfaces sampled for the tests performed. In the example, the coefficients in the lower level of detail of the code (levels 0, 1, and 2) present very similar results, owing to the average values of *R*_*a*_. This is noticeable because both the *R*_*a*_ in the first column and the values *R*_*a*_*min* and *R*_*a*_*max* in the second and third columns of the table are very similar (even the images are very similar). However, in the third level of division (level 3), the difference between the surfaces is better perceived. The images have a larger difference and the *R*_*a*_*min* and *R*_*a*_*max* have a greater difference compared to the results of previous levels.

The engineering professional, however, can use the level of subdivision that best fits his purpose, because it is possible to search for a pattern of similarity among blocks or to analyze their differences in more detail.

#### 6.1.2 Comparative roughness assessment

The comparison of results obtained at different temperatures indicates to the engineering professional a metric to determine which process to adopt, according to the desired roughness level. Table 1 presents the results (*R*_*a*_*avg*, *R*_*a*_*min*, *R*_*a*_*max* e *R*_*a*_*adv*), obtained by comparing coefficients on all surfaces of each block and also on all surfaces of all. the blocks of the same temperature group (same fire peak). The values show that the samples in the firing temperature group of ${1000}^{\circ }$C show greater variation of coefficients as well as greater roughness value, indicating that this firing temperature presents greater roughness along the surface and, consequently, generates a shear strength^2^.

**Table 1:** Surface roughness comparison by temperature group.

Temperature group	All blocks	1 block	2 block	3 block
Blocks of 800 °C	${R}_{a}avg=3.457\,\mu m$ ${R}_{a}min=2.169\,\mu m$ ${R}_{a}max=8.134\,\mu m$ ${R}_{a}sdv=0.917\,\mu m$	${R}_{a}avg=3.575\,\mu m$ ${R}_{a}min=2.214\,\mu m$ ${R}_{a}max=7.978\,\mu m$ ${R}_{a}sdv=1.026\,\mu m$	${R}_{a}avg=3.348\,\mu m$ ${R}_{a}min=2.219\,\mu m$ ${R}_{a}max=7.980\,\mu m$ ${R}_{a}sdv=0.802\,\mu m$	${R}_{a}avg=3.448\,\mu m$ ${R}_{a}min=2.169\,\mu m$ ${R}_{a}max=8.134\,\mu m$ ${R}_{a}sdv=0.918\,\mu m$
Blocks of 1000 °C	${R}_{a}avg=3.502\,\mu m$ ${R}_{a}min=1.814\,\mu m$ ${R}_{a}max=15.780\,\mu m$ ${R}_{a}sdv=1.026\,\mu m$	${R}_{a}avg=3.262\,\mu m$ ${R}_{a}min=2.049\,\mu m$ ${R}_{a}max=10.037\,\mu m$ ${R}_{a}sdv=0.712\,\mu m$	${R}_{a}avg=3.261\,\mu m$ ${R}_{a}min=1.986\,\mu m$ ${R}_{a}max=9.966\,\mu m$ ${R}_{a}sdv=0.792\,\mu m$	${R}_{a}avg=3.984\,\mu m$ ${R}_{a}min=1.814\,\mu m$ ${R}_{a}max=15.780\,\mu m$ ${R}_{a}sdv=1.478\,\mu m$

First column are showed values computed from all blocks in the same temperature group. The other columns shows the mean (*R*_*a*_*avg*), minimum (*R*_*a*_*min*), maximum (*R*_*a*_*max*) and standard deviation (*R*_*a*_*sdv*) values for each block.

**Figure 12:**
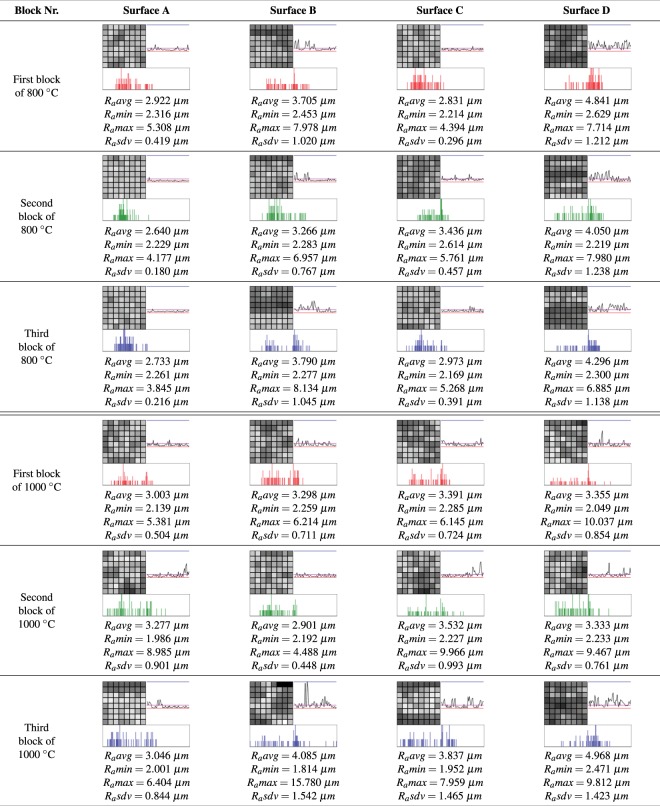
All results.

The other form of analysis used in this work is the comparison of data through the newly proposed analysis tools. Combining the use of the three tools, several behaviors, patterns, and analyses of the sampled surfaces can be inferred. Figure 12 presents the results obtained in the tests performed for the model validation.

For computing the signatures, the minimum (*R*_*a*_*min*), maximum (*R*_*a*_*max*), and average (*R*_*a*_*avg*) values were computed on all the sampled surfaces. The values found were: *R*_*a*_*min* = 1.714 *μm*, *R*_*a*_*max* = 15.78 *μm*, and the average roughness *R*_*a*_*avg* was 3.484 *μm*.

The roughness signature assists in the evaluation of roughness and interface adhesion in different temperature samples, because, notably (see Fig. 12), there is a differentiated behavior between blocks of different firing temperatures. As shown in Fig. 12, some surfaces have greater roughness variation than others. This is perceived by the color variation of the signature. However, other behaviors can also be identified. For example, in the tests performed, it was possible to verify a standardized behavior (similarity) between the surfaces of similar regions of all the blocks, see column “Surface A” of Fig. 12. It is verified that in this region of the blocks, the coefficients show generally low variation; they have values near or below the average roughness (*R*_*a*_*avg*). This behavior is highlighted by the coefficient graph.

Another tool used in the evaluation of results, the histogram, allows us to compare the variation of roughness values between blocks of different firing temperatures. It is generally noted that the surfaces of the blocks with a firing temperature of 800 °C have concentrated the coefficients closest to the center of the histogram (or closer to the value *R*_*a*_*avg*). On the surfaces of the blocks of 1000 °C, there is a greater variation or dispersion of the values in relation to the average roughness (*R*_*a*_*avg*).

Through the results presented with the tools used for analysis, it was possible to quantitative and visually verify that the blocks with a firing temperature of 1000 °C have greater variation of roughness compared to the blocks with a firing temperature of 800 °C, which supposes an effect of favoring the interfacial adhesion with concrete.

Finally, the proposed tools allow a greater variation of the evaluation criteria of surface roughness in relation to the quantitative form presented in the reference works^6,11^ and the subjective methods of^15–18,21,22^. As it can be verified in the presented results, it is possible to analyze in several levels of detail, allowing comparisons and assumptions that are not easily determined by the simple analysis of global coefficients.

## Conclusion

The evaluation of the surface quality of materials through the measurement and analysis of roughness parameters is known^6,11,15^ to be an effective way of determining the quality or standardization of surfaces. In addition, the possibility of computing the results in a localized way and through the spatial division and hierarchical organization and subdivision of these locations provides civil engineering professionals a more accurate control tool for the comparison and evaluation of surface quality. For example, it is possible to submit several surface samples of the same material and to check the patterns of roughness of the samples, as well as to verify and evaluate the distortions due to the way the piece was constructed or environmental factors such as temperature, pressure, and friction.

The proposed tools are effective for the analysis and evaluation of roughness following the concepts defined in the reference works^6,11^, and bring a new and advantageous perspective on the analysis of surface roughness, as they allow a localized and detailed assessment of roughness coefficients of surfaces and at the same time facilitate comparative analysis among different sample surfaces.

Although this work presents a method for surface roughness computation applied to the civil engineering context, it can be successfully used in other contexts as well. For the study of rock mass formations in geomechanics and geodynamics of rock masses, the roughness analysis is important to determine shear strength, deformation and seepage behaviors of rock surfaces discontinuities. Several works such as^19,36,37^ point out the difficulty of data analysis by the traditional method and the tendency of using 3D point clouds to calculate surface roughness with efficiency and precision. As described in^19,36,37^ the roughness is a part of the calculation performed for shape measurement of a surface profile. In the context of rock geomechanics, the JRC (Joint Roughness Coefficient) parameter is used. This parameter is calculated based on the surface geometry and basically is a measure related to the distance of the point to the fitting plane of the surface, similar to the *R*_*a*_ computed in this work. Poropat^36^ further describes that roughness can affect shear strength at various scales, both at waviness and at micro-roughness levels. That the characterization of roughness must be understood and is associated with the scale. The proposed method in this work can be used for multi-scale roughness computation, as it enables the analysis of roughness at various levels of hierarchical representation. In addition, the proposed roughness analysis tools help in understanding the roughness patterns, quantitatively and visually indicating texture variation behavior along the surface.

Finally, there are still several points that can be explored to try to improve the general evaluation of surfaces, such as data acquisition by photogrammetry and the evaluation of the area of the rough part, not just the height, this would indicate with more precision the adhesion that each surface can allow.

## Data Availability

The datasets that were generated and/or analysed during the current study are freely available from the corresponding author on a request.

## References

[1] Thamboo JA, Dhanasekar M (2015). Characterisation of thin layer polymer cement mortared concrete masonry bond. Constr. Build. Mater..

[2] Venkatarama Reddy B, Lal R, Nanjunda Rao K (2007). Enhancing bond strength and characteristics of soil-cement block masonry. J. materials civil engineering.

[3] Taha, M. R. & Shrive, N. The use of pozzolans to improve bond and bond strength. In *9 th Canadian masonry symposium. Canadá* (2001).

[4] Myshkin N, Petrokovets M, Chizhik S (1998). Simulation of real contact in tribology. Tribol. Int..

[5] Marshall SJ, Bayne SC, Baier R, Tomsia AP, Marshall GW (2010). A review of adhesion science. dental materials.

[6] Santos PM, Júlio EN (2013). A state-of-the-art review on roughness quantification methods for concrete surfaces. Constr. Build. Mater..

[7] Perez F, Bissonnette B, Courard L (2009). Combination of mechanical and optical profilometry techniques for concrete surface roughness characterisation. Mag. Concr. Res..

[8] Klingvall ER, Rännar L-E, Bäckstöm M, Carlsson P (2016). The effect of ebm process parameters upon surface roughness. Rapid Prototyp. J..

[9] Garbacz, A., Courard, L. & Kostana, K. Characterization of concrete surface roughness and its relation to adhesion in repair systems. *Mater. Charact*. 56, 281–289, 10.1016/j.matchar.2005.10.014, *9th ECSIA and 7th STERMAT: Stereology and Image Analysis in Materials Science* (2006).

[10] Sadowski, Ł., Czarnecki, S. & Hoła, J. Evaluation of the height 3d roughness parameters of concrete substrate and the adhesion to epoxy resin. *Int. J. Adhesion Adhesives* **67**, 3–13, 10.1016/j.ijadhadh.2015.12.019, *Special Issue on Adhesion, Surface Preparation and Adhesive Properties* (2016).

[11] Moreau, N., Roudet, C. & Gentil, C. Study and Comparison of Surface Roughness Measurements Journées du Groupe de Travail en Modélisation Géométrique (GTMG’14), Lyon (2014).

[12] Miro MM (2015). 3d and 2d structural characterization of 1d al/al2o3 biphasic nanostructures. J. microscopy.

[13] Grigoriadis K (2016). Use of laser interferometry for measuring concrete substrate roughness in patch repairs. Autom. Constr..

[14] Stolz CM, Masuero AB (2015). Analysis of main parameters affecting substrate/mortar contact area through tridimensional laser scanner. J. colloid interface science.

[15] Berger, M. *et al*. State of the art in surface reconstruction from point clouds. In *Eurographics star reports*, vol. 1, 161–185 (2014).

[16] Kazhdan, M., Bolitho, M. & Hoppe, H. Poisson surface reconstruction. In *Proceedings of the Fourth Eurographics Symposium on Geometry Processing*, SGP ’06, 61–70 (Eurographics Association, Aire-la-Ville, Switzerland, Switzerland, 2006).

[17] Kazhdan M, Hoppe H (2013). Screened poisson surface reconstruction. ACM Transactions on Graph. (ToG).

[18] Taubin, G. Smooth signed distance surface reconstruction and applications. *Prog. Pattern Recognition, Image Analysis, Comput. Vision, Appl*. 38–45 (2012).

[19] Mah J, Samson C, McKinnon SD, Thibodeau D (2013). 3d laser imaging for surface roughness analysis. Int. J. Rock Mech. Min. Sci..

[20] Lee, C. H., Varshney, A. & Jacobs, D. W. Mesh saliency. In *ACM SIGGRAPH 2005 Papers, SIGGRAPH ’05*, 659–666, 10.1145/1186822.1073244 (ACM, New York, NY, USA, 2005).

[21] Schall, O. & Samozino, M. Surface from scattered points. In *A Brief Survey of Recent Developments. 1st International Workshop on Semantic Virtual Environments*, Page S, 138–147 (2005).

[22] Nan, L. & Wonka, P. Polyfit: Polygonal surface reconstruction from point clouds. In *The IEEE International Conference on Computer Vision (ICCV)* (2017).

[23] Wang K, Torkhani F, Montanvert A (2012). A fast roughness-based approach to the assessment of 3D mesh visual quality. Computers and Graphics.

[24] Labatut, P., Pons, J. & Keriven, R. Efficient multi-view reconstruction of large-scale scenes using interest points, Delaunay triangulation and graph cuts. In *2007 IEEE 11th International Conference on Computer Vision*, 1–8, 10.1109/ICCV.2007.4408892 (2007).

[25] Zhou, Y., Shen, S. & Hu, Z. Detail preserved surface reconstruction from point cloud. *Sensors* **19**, 10.3390/s19061278 (2019). 10.3390/s19061278 PMC6471080 30871277

[26] Wang, W. *et al*. Surface reconstruction from unoriented point clouds by a new triangle selection strategy. *Computers and Graphics*, 10.1016/j.cag.2019.08.002 (2019).

[27] Bolitho, M., Kazhdan, M., Burns, R. & Hoppe, H. Multilevel streaming for out-of-core surface reconstruction. In *Symposium on geometry processing*, 69–78 (Citeseer, 2007).

[28] Bolitho, M., Kazhdan, M., Burns, R. & Hoppe, H. Parallel poisson surface reconstruction. In International symposium on visual computing, 678–689 (Springer, 2009).

[29] Wang R, Peethambaran J, Chen D (2018). Lidar point clouds to 3-d urban models: a review. IEEE J. Sel. Top. Appl. Earth Obs. Remote. Sens..

[30] Gadelmawla E, Koura M, Maksoud T, Elewa I, Soliman H (2002). Roughness parameters. J. Mater. Process. Technol..

[31] Sander, M. *A Practical Guide to the Assessment of Surface Texture* (Mahr Feinprüf, 1991).

[32] Mummery, L. *Surface Texture Analysis: The Handbook* (Hommelwerke GmbH, 1992).

[33] Thomas, T. *Rough Surfaces* (Imperial College Press, 1999).

[34] Lavoué, G. A roughness measure for 3d mesh visual masking. In *Proceedings of the 4th symposium on Applied perception in graphics and visualization*, 57–60 (ACM, 2007).

[35] Lavoué G (2009). A local roughness measure for 3d meshes and its application to visual masking. ACM Trans. Appl. Percept..

[36] Poropat, G. Measurement of surface roughness of rock discontinuities. *Rock Eng. Difficult Cond*. (2009).

[37] Ge Y (2015). A description for rock joint roughness based on terrestrial laser scanner and image analysis. Sci. reports.

